# Cyclic photochemical re-growth of gold nanoparticles: Overcoming the mask-erosion limit during reactive ion etching on the nanoscale

**DOI:** 10.3762/bjnano.4.100

**Published:** 2013-12-12

**Authors:** Burcin Özdemir, Axel Seidenstücker, Alfred Plettl, Paul Ziemann

**Affiliations:** 1 Institute of Solid State Physics, Ulm University, D-89069 Ulm, Germany

**Keywords:** Au nanoparticles, block copolymer micellar lithography, photochemical growth, reactive ion etching, self-assembly

## Abstract

The basic idea of using hexagonally ordered arrays of Au nanoparticles (NP) on top of a given substrate as a mask for the subsequent anisotropic etching in order to fabricate correspondingly ordered arrays of nanopillars meets two serious obstacles: The position of the NP may change during the etching process and, thus, the primary pattern of the mask deteriorates or is completely lost. Furthermore, the NP are significantly eroded during etching and, consequently, the achievable pillar height is strongly restricted. The present work presents approaches on how to get around both problems. For this purpose, arrays of Au NPs (starting diameter 12 nm) are deposited on top of silica substrates by applying diblock copolymer micelle nanolithography (BCML). It is demonstrated that evaporated octadecyltrimethoxysilane (OTMS) layers act as stabilizer on the NP position, which allows for an increase of their size up to 50 nm by an electroless photochemical process. In this way, ordered arrays of silica nanopillars are obtained with maximum heights of 270 nm and aspect ratios of 5:1. Alternatively, the NP position can be fixed by a short etching step with negligible mask erosion followed by cycles of growing and reactive ion etching (RIE). In that case, each cycle is started by photochemically re-growing the Au NP mask and thereby completely compensating for the erosion due to the previous cycle. As a result of this mask repair method, arrays of silica nanopillar with heights up to 680 nm and aspect ratios of 10:1 are fabricated. Based on the given recipes, the approach can be applied to a variety of materials like silicon, silicon oxide, and silicon nitride.

## Introduction

Nanoparticles (NP), though primarily sought-after because of their new size- and shape-dependent physical or chemical properties, also play an important role in the context of nanolithography. For this purpose, NP that are deposited onto a given substrate are applied as masks during the subsequent anisotropic etching processes such as reactive ion etching (RIE). In this way, the original pattern of NP positions is transferred into the subjacent substrate and, finally, an array of nanopillars is obtained by forming a replica of the original NP pattern.

To develop this idea into a flexible nanolithographic tool, a number of additional requirements should be fulfilled:

1) The control over the size of the NP with size distributions as narrow as possible.

2) An adequate etching resistance of the NP.

If randomly positioned nanopillars of homogeneous size and shape meet the pre-requisites for a specific study, the above two requirements are already sufficient. In quite a number of cases, however, from both, a fundamental as well as an application point of view, periodically arranged nanopillars would be highly desirable. This is related to the fact that suitably functionalized nanopillars may act as emitters of, e.g., electrons or photons or, alternatively, as scattering or pinning centers. In all these situations a periodicity of the pillars may lead to interesting interference effects. On the other hand, such a constraint demands for additional preparational requirements on the NP serving as masks:

3) The periodic arrangement of NP, if possible of various symmetries like hexagonal or square symmetry.

4) The control over the interparticle distance within a given periodic arrangement.

5) The maximizing of defect-free domain sizes of such NP lattices.

A relative simple and affordable approach that nevertheless addresses all the above requirements is based on the self-organization of organic carrier systems such as colloids or reverse micelles. The underlying idea is to form such carriers in a suitable solution and to load them with metal precursors. During their deposition onto a given substrate by dip-coating the carriers self-organize into highly ordered hexagonal arrays, which define the position of the metal NP prepared in the next step. For that purpose, the organic matrix is completely removed by exposure to oxygen and hydrogen plasmas while, in parallel, the precursors are reduced into the metallic state to form NP. The reliability of the approach has been demonstrated previously for colloidal [1–2] as well as micellar [3–6] carriers. In the following we focus exclusively on Au-precursor (HAuCl_4_) loaded diblock-copolymers [poly(styrene)(PS)-*block*-poly(2-vinylpyridine)(P2VP)], which is commercially available from Polymer Source Inc. Canada] that form reverse micelles in toluene. Details on preparing the solution, the dip-coating as well as the plasma processes can be found in [6]. Thus, in the present context it may suffice to describe how the method, often addressed as block copolymer micelle lithography (BCML), meets the above requirements.

For a given copolymer like PS(1850)-*b*-P2VP(900) in toluene (the monomer numbers given in parentheses are a measure of each block length) the size (diameter) of the finally obtained Au NP are completely determined by the amount of precursor salt added to the solution. By prolonged stirring, the micelles will be homogeneously loaded with precursor to deliver Gaussian size distributions of the final Au NP. Experimentally, a size window of 2 nm ≤ diameter ≤ 12 nm for Au NP can be realized if the monomer number especially of the P2VP block is properly adjusted [6]. Thus, size control as required in 1) above is principally given by the micellar approach. In practice, however, with such Au NP that serve as etching masks only the largest ones with diameters of 12 ± 1.5 nm turned out useful. This is at least partly related to requirement 2), their etching resistance. For a given purpose like etching a specific material by applying a recipe optimized with respect to etching rate and/or shape of the resulting walls, rather than giving the absolute etching rate of a mask, the selectivity of the process is most important. In the present study with its emphasis on the anisotropically etching of SiO_2_ substrates by using Au NP masks, the selectivity may be quantified by the ratio of etching rates *r*_SiO2_/*r*_Au_, which is experimentally found to be around 5.3. Thus, for our largest Au NP of 12 nm nanopillars with an approximate maximum height of only 64 nm could be expected. On the other hand, the micellar approach fulfills the above additional requirements 3) and 4): Self-organization of the precursor-loaded micelles during their deposition onto the substrate to form hexagonal arrays leads to correspondingly arranged Au NP after plasma and annealing treatments [4–6]. Furthermore, the total length of the diblock-copolymer and the pulling velocity of the substrate during the dip-coating process [7] determine the interparticle distance that is finally obtained. In this way, distances between 30 and 140 nm can be controllably realized. Even significantly larger interparticle distances can be accomplished by combining the micellar method with standard e-beam or photo lithography. In that case, also the symmetry of the NP array can be changed from hexagonal to, e.g., square [8]. Size and distance of the NP are, however, not completely uncorrelated. Rather, larger diameters require larger lengths of the P2VP block, a parameter which has an upper limit to avoid uncontrolled conformational changes. As a consequence, the above mentioned diameter of 12 nm for Au NP already represents the upper limit in size, which immediately leads to the corresponding upper limits for the geometry of etched SiO_2_ nanopillars: Diameter 12 nm and height 64 nm. Thus, if these absolute geometry values are not sufficient and larger values are necessary for one or both parameters, the question arises whether such requirements can still be accomplished on the basis of a micellar approach, which specifically offers highly ordered arrays of NP. It is the focus of the present work to present a solution to this problem by a selective re-growth of Au NP, which were already eroded by a previous etching step. However, before describing details of our approach, alternative methods which have been reported previously in the literature will be introduced for comparison.

Instead of using metallic NP as a mask for subsequent etching, nanoimprint lithography has been suggested and successfully demonstrated for preparing Si pillar arrays. For that purpose, a tri-layer imprint stack is prepared on top of a Si wafer and the sought pattern is transferred by a pre-fabricated mold imprinted into the top polymer layer followed by deep reactive ion etching (an optimized Bosch process) [9]. In this way, 200 nm-pitch patterns of 50 nm Si pillars with heights of up to 2.4 µm were prepared. These heights profit from the larger etching rates of Si as compared to the patterned SiO_2_ substrates. A method based on the self-organization of diblock-copolymers and, thus, closely related to the present approach, has been reported by Krishnamoorthy et al. [10]. Rather than NP these authors directly applied PS-*b*-P2VP reverse micelles as nano-masks. Due to the low etching resistance of these masks, however, an intermediate SiO_2_ layer had to be pre-patterned first to obtain a more resistant mask for the subsequent transfer of the pattern into the subjacent substrate. The self-organization of PS-*b*-PMMA (polymethylmethacrylate) diblock-copolymers forms hexagonally arranged cylindrical PMMA nanodomains that are surrounded by PS. Selective removal of the PMMA nanodomains by an oxygen plasma process delivers the mask for subsequent SiO_2_ etching. In this way, densely packed silica nanopillars with a diameter of 20 nm and a height of 150 nm could be fabricated [11]. Still another variant of exploiting the self-assembly of diblock-copolymers was reported recently by Ghoshal et al. [12]. It is based on the microphase separation of PS-*b*-PEO that forms hexagonally arranged cylindrical PEO (poly-ethylene oxide) domains, which after chemical activation, can be loaded with inorganic components like metal cations. By injecting iron(III) nitrate into the PEO domains and applying a UV/ozone treatment iron oxide is obtained while the organic components are removed. Thus, after an additional annealing, a hexagonal array of Fe_2_O_3_ particles is obtained and can be used as mask for a subsequent etching process. Both of the last two approaches suffer from the restricted flexibility of changing the inter-domain distance as well as their size with immediate consequences on the range of mask geometries.

## Results and Discussion

As mentioned above, the basic idea of the present work is to use ordered arrays of Au nanoparticles prepared on top of silica substrates via a micellar route and to overcome mask erosion during the subsequent etching processes by cyclic re-growth of the Au NP. Thus, two important issues had to be addressed: The choice of a suitable selective re-growth recipe and a test whether its application conserves the lateral position of the seeded Au NP. An electroless Au deposition onto already existing Au seeds, which is based on combining a gold salt (HAuCl_4_) and a reducing agent (NH_2_OH), has been reported [13–14]. Recently, a direct photochemical re-growth of Au particles without any reducing agents was developed [15–16]. This method was tested here in combination with Au NP fabricated by the micellar technique. It turned out that in this way particles with starting diameters of 12 nm could be enlarged up to 30 nm while keeping the relative width of their size distributions as well as their lateral positions. For enlargements beyond 30 nm, however, the original hexagonal order of the particle array is lost and the size distribution significantly broadens. In order to avoid these detrimental effects and to allow size enhancements at least up to 50 nm, an additional stabilizing layer was introduced as described in the following.

**Figure 1:**
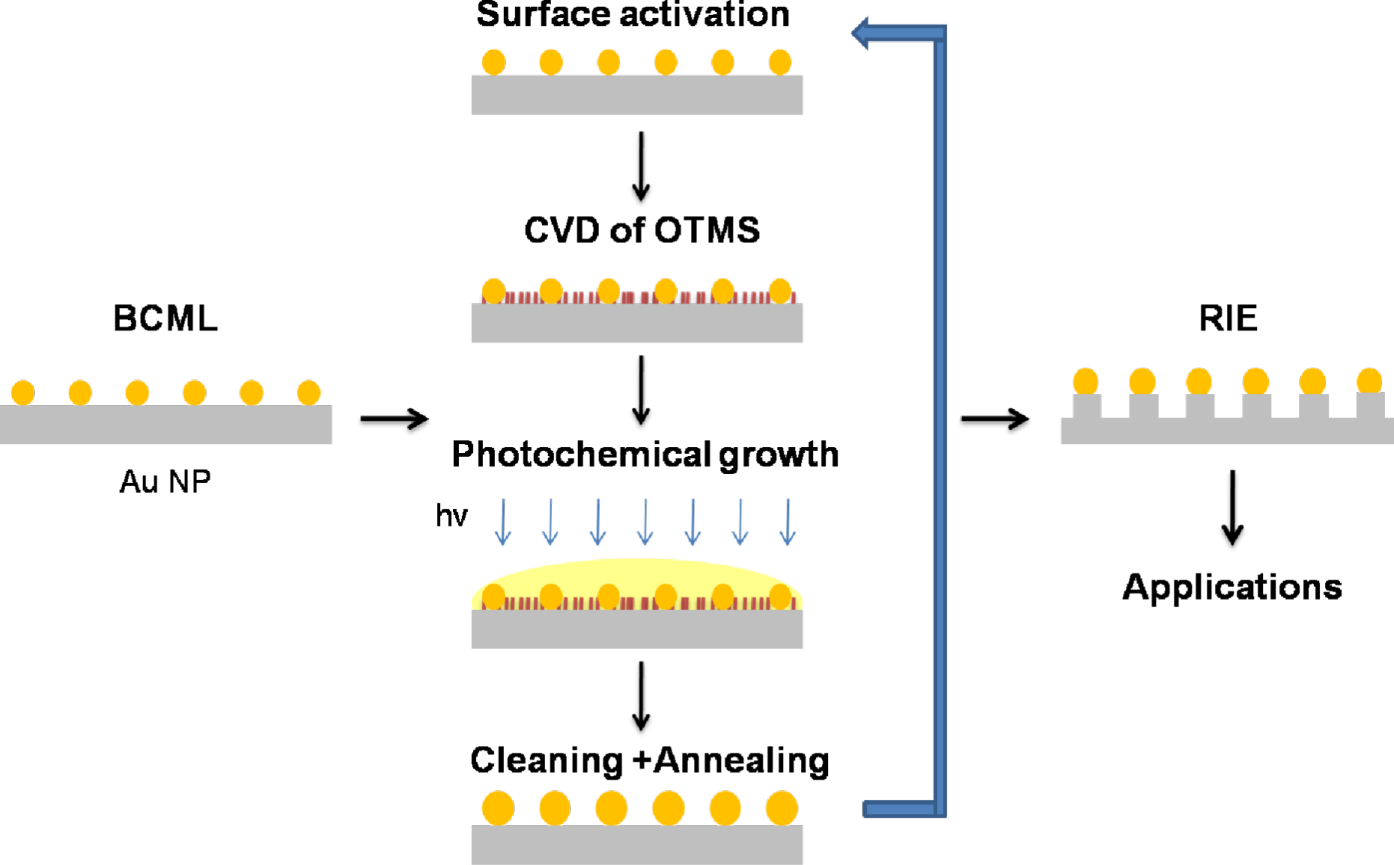
Schematics of nanopillar fabrication by applying Au NP as masks after fine tuning their size by an UV-light activated photochemical process. To stabilize the NP positions during the growth process, an OTMS layer is deposited by CVD first. This layer selectively forms chemical bonds to the silica substrate while leaving the Au NP uncovered.

Alkyltrimethoxysilanes are known to form self-assembled monolayers (SAM) on substrates after immersion into the corresponding solutions and were reported to act stabilizing on the location of nanoparticles [17–18]. Following this idea, in the present work octadecyltrimethoxysilane (OTMS) SAMs were prepared on top of the previously fabricated Au NP by chemical vapor deposition (CVD) rather than immersion of the silica substrates into OTMS solutions. Like immersion the much faster CVD method results in selective reactions of the methoxysilane functional groups with the silanol groups of the SiO_2_ surface. Consequently, OMTS bonding will not occur on the Au NP, which leaves their surfaces uncoated and, thus, amenable to subsequent electroless re-growth processes. The complete process is presented in Figure 1 and more information is given in the section Experimental further below. In short, the Au NP prepared by BCML on silica are stabilized by CVD-coating the substrate with OMTS. The success of this coating can be easily tested by contact angle measurements: The hydrophilic silica substrate changes its starting angle from almost zero to 104° due to the hydrophobic OTMS coating. The second process step is the deposition of a 5 mM HAuCl_4_ solution and its exposure (typically for 6 min) to UV-radiation in order to accomplish the selective growth of the Au NP. The substrates are cleaned in acetone and isopropanol baths, followed by drying with nitrogen. After the subsequent annealing of the grown NP in order to re-establish their spherical shape, the growth process can be repeated as indicated by the arrow in Figure 1 until the desired size of the NP is obtained. Details of the annealing process are given in the Experimental part. Finally, reactive ion etching completes the fabrication of hexagonally arranged nanopillars.

**Figure 2:**
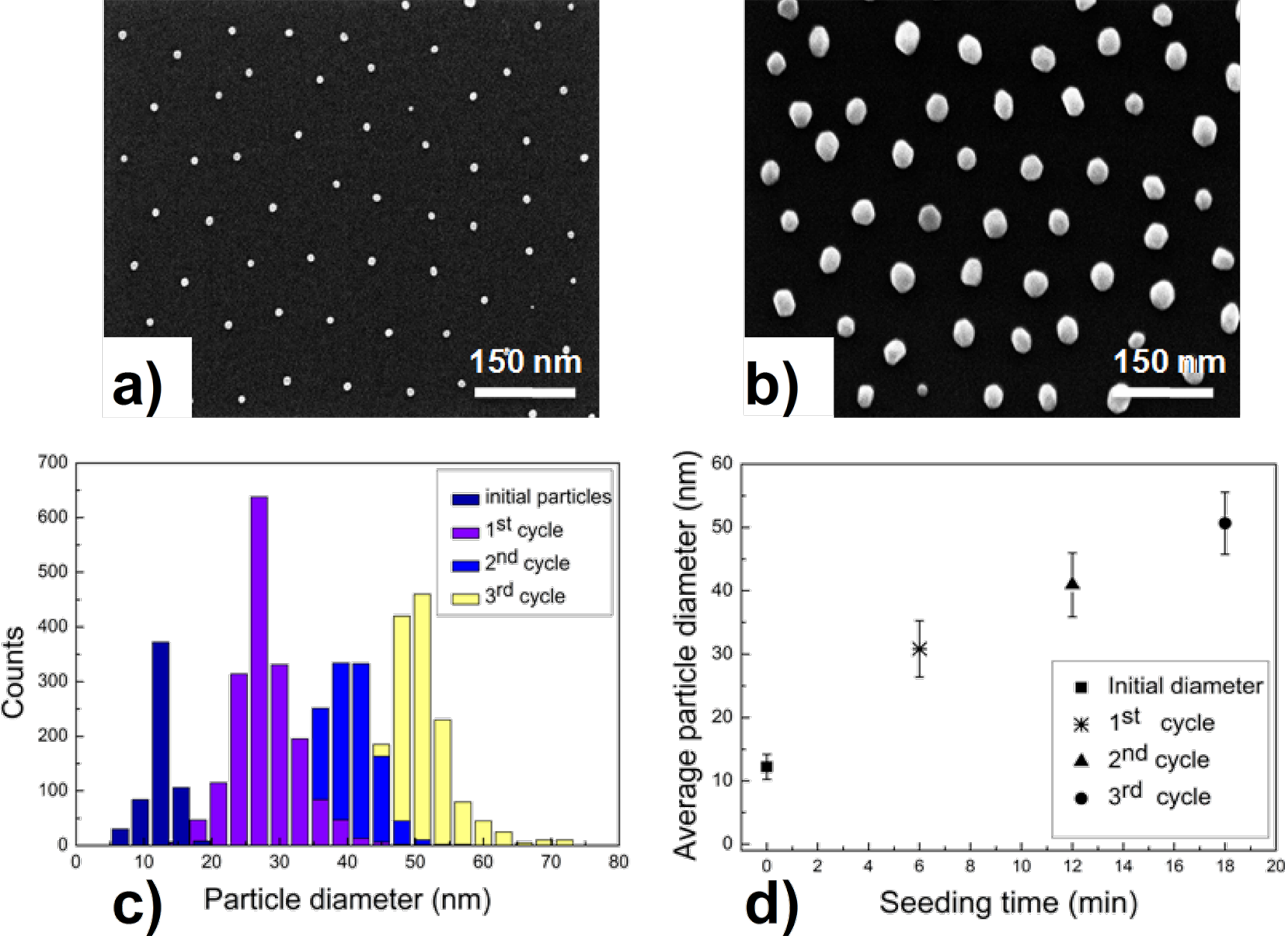
HRSEM images of (a) as prepared Au NP on silica (average diameter 12 nm), (b) Au NP after the second electroless size-enhancement cycle (average diameter 40 nm). Panel (c) presents the size distributions of the seeding steps defined in panel (d), which includes the corresponding seeding times.

The option to multiply repeat the growth process turned out to be indispensable since the OTMS layer strongly deteriorates under UV exposure and loses its stabilizing property as demonstrated in Figure S1 (Supporting Information File 1). As a consequence, the growth of Au NP from their starting diameter of 12 nm up the final value of 50 nm is subdivided into three steps as will be shown in Figure 2. There, the HRSEM image in panel a) represents the starting size (12 nm) and arrangement of Au NP, while that in panel b) characterizes the state after NP growth up to 40 nm. These images provide clear evidence for the conserved hexagonal order of the NP and, thus, for the stabilizing effect of the OTMS layer (cf. result without OTMS as given in Figure S1 in the Supporting Information File 1). To prove the influence of the successive growing processes on the position stability of the NP, two HRSEM images taken from exact the same location before and after each experiment were compared. For this purpose the surface of the substrate was marked by a diamond tip to observe the changes within a defined lateral window. For accurate analysis, the shift and rotation of the SEM images were taken into account as well and details are given in the Section S2 and Figure S2 (Supporting Information File 1). One finds that NP that were increased in size as described above retain their positions; only very few particles get completely lost (< 3%). Also, in both SEM images of Figure 2, the homogeneity of the size distrubutions is immediately visible. This point is made more quantitative in panel c) where such distributions are presented from the starting and final state as well as for two more intermediate growth states as indicated in panel d) giving the related average NP diameter as a function of the corresponding seeding times.

**Figure 3:**
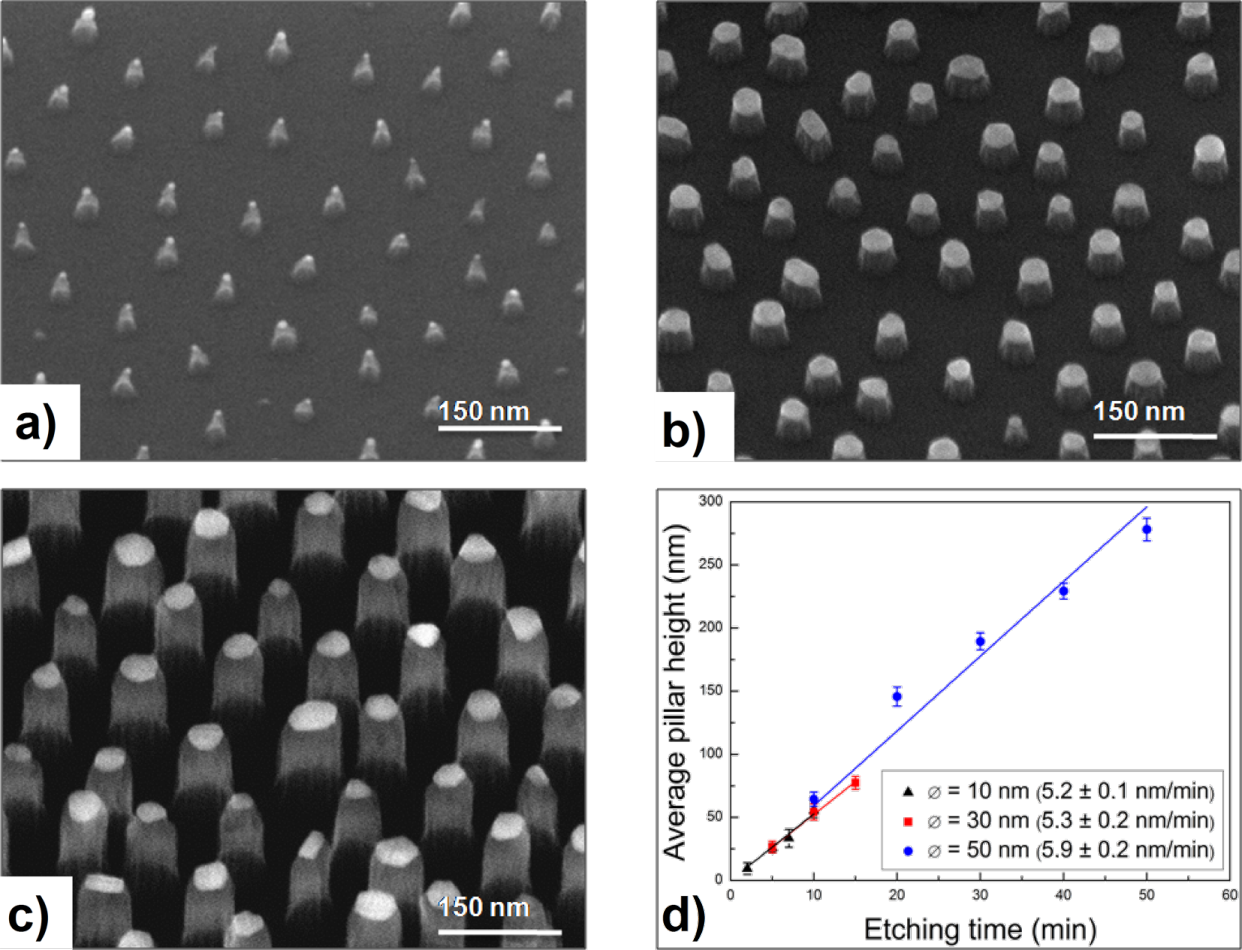
Nanopillar arrays etched into silica applying Au NP of different diameters as masks: (a) NP diameter = 12 nm, pillar height = 50 nm (averages), (b) NP diameter = 30 nm, pillar height = 50 nm (averages), (c) NP diameter = 50 nm, pillar height = 190 nm (averages). Panel (d) Development of the average pillar height as a function of etching time for the three differently sized Au NP masks.

The final step of fabricating arrays of nanopillars applying differently sized Au NP as masks is documented in Figure 3 for three starting diameters: 12 nm, 30 nm, 50 nm. The HRSEM images (substrates tilted by 30°) presented in panels a)–c) represent intermediate states of etching sequences introduced in panel d), thus in all cases mask residuals are still visible. These images indicate that the Au NP masks keep their position during etching, i.e. the pattern formed by the nanopillars directly reflects the original mask pattern. Furthermore, panel d) confirms the expected linear time dependence of the resulting average pillar height revealing an average etching rate of 5.5 nm/min for SiO_2_ practically independent of the mask diameter. This rate is accomplished by a RIE-process optimized for etching Si-materials (Si, Si_3_N_4_, and SiO_2_) on the nanoscale (cf. Experimental). The lifetime of the masks, however, obviously depends on their size, the smaller the shorter. This has an immediate effect on the shape of the resulting nanopillars: Small 12 nm particles create more conical shaped pillars (sidewall angle < 70°), whereas larger ones cause rather cylindrical shapes (sidewall angle > 80°) in the case of SiO_2_, which can be further improved in the case of Si (sidewall angle > 85°).

As mentioned already in the introduction, the etching contrast between SiO_2_ and Au NP that was found experimentally is approximately 5.3. Thus, at etching times of around 50 min, which correspond to a pillar height of about 270 nm, even the 50 nm Au masks are eroded too strongly to conserve the cylindrical pillar shape. For some applications however, such as the determination of cell–surface interaction, the pillar height is more important than its shape [19]. But even then, for 50 nm Au NP the maximum pillar height is still restricted to less than 300 nm. In terms of achievable aspect ratios the above height restriction translates into 3:1 for small NPs and up to 5:1 for large NPs on SiO_2_.

**Figure 4:**
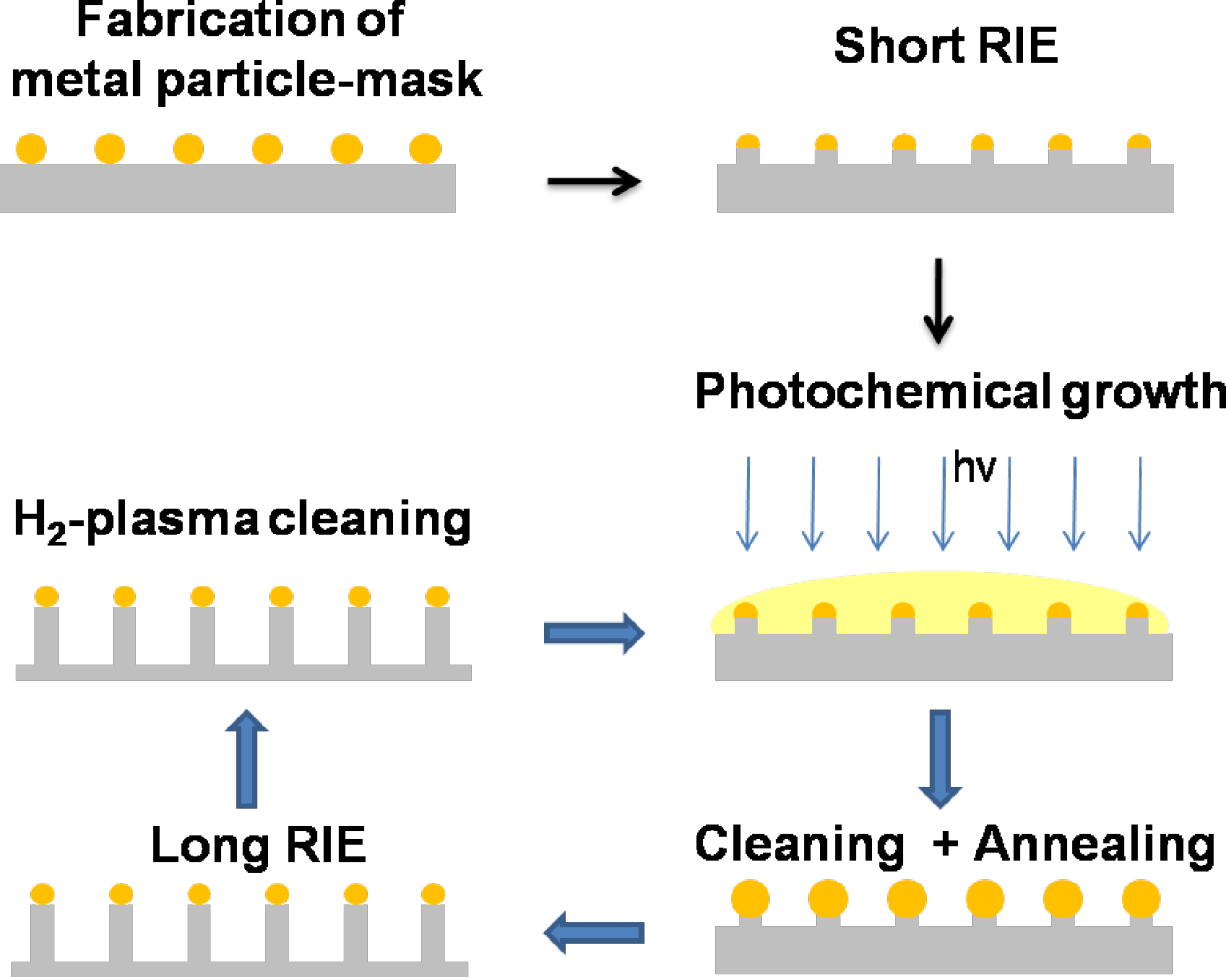
Schematics of fabricating nanopillars by applying Au NP as masks, which are repeatedly re-grown by an UV light activated photochemical process thus compensating their erosion due to previous etching.

The above method to obtain almost cylindrical SiO_2_ nanopillars (sidewall angle > 80°) is based on the idea of stabilizing and re-growing the original Au NP etching masks, while the final etching is performed in one step. An alternative route is introduced in Figure 4.

**Figure 5:**
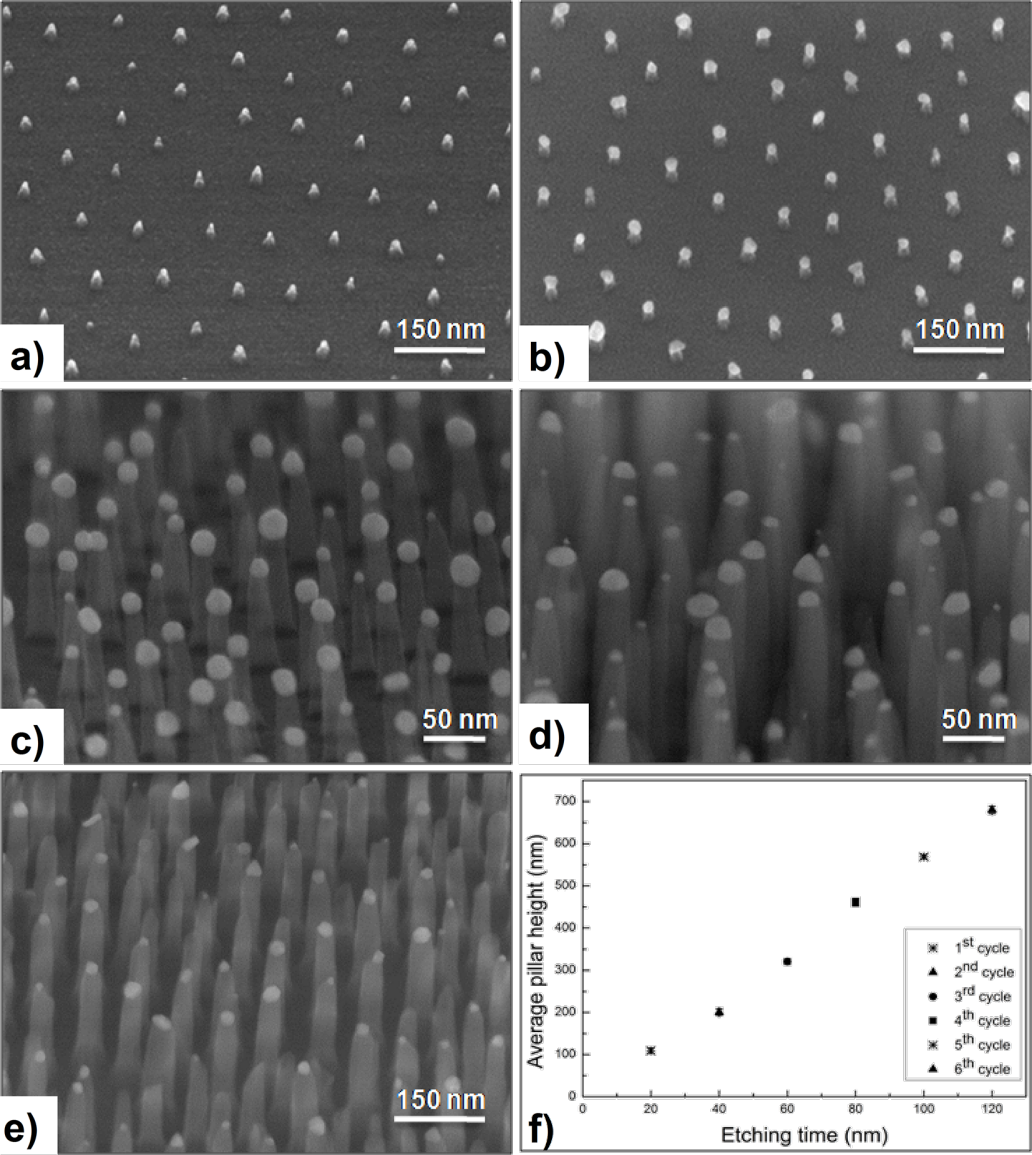
HRSEM images [(a), (b), (e) substrates tilted by 30°, (c), (d) substrates tilted by 80°] of nanopillar arrays with various heights as obtained by the approach outlined in Figure 4. (a) Pillar pattern fabricated on the SiO_2_ substrates by a short RIE step [pillar height (*h*) = 25 nm, initial NP diameter = 12 nm, final NP diameter = 9 nm (average values)]. (b) Photochemically grown Au particles on the seeds of panel (a) [final NP diameter = 24 nm (average)]. (c) Seeded Au NP on 100 nm pillars after the 1st etching cycle and (d) residual Au masks after the 2nd etching cycle, pillar height 205 nm (average). (e) Residual Au masks after the 3rd etching cycle, pillar height 320 nm and aspect ratio 8:1 (average). (f) Average pillar height as a function of the total RIE time. Detailed numbers and HRSEM images related to this sequence are given in Figure S3 and Table S1 (Supporting Information File 1).

In this approach, rather than applying an additional stabilizing OTMS layer, the original Au NP are exposed to a RIE process short enough that erosion of the NP can be neglected. As a result, one obtains correspondingly short nanopillars that are still capped by Au NP. Subsequent photochemical growth combined with a cleaning and annealing step delivers enlarged Au caps, which serve as masks during the subsequent long RIE process. After interrupting the etching and H_2_-plasma cleaning, the Au NP are renewed by a photochemical re-growth that compensates for their erosion due to the previous etching. This mask renewal can be applied periodically as indicated by the arrow loop in Figure 4. Since the re-growth process selectively occurs at the Au mask, the original NP pattern is conserved. Furthermore, since the number of re-growth cycles is not limited, the previous height restriction can be overcome. Actually, the maximum height now depends on the slope of the pillar walls. For conical pillars the maximum is reached when the pillar bases start to overlap. The more perfect their cylindrical shape the higher the pillars and the larger the aspect ratio. A corresponding experimental result is presented in Figure 5. In it the HRSEM images document various etching stages: The short (25 nm) pillars produced by the short starting RIE are shown in panel a). The bright residual Au caps, which form the seed particles for the subsequent photochemical re-growth processes are clearly visible. Panel b) shows the result of the first re-growth step on top of the pillars from panel a). The further etching progress is presented in panels c)–e) and the associated linear relation between total pillar height and etching time is summarized in f). In accordance with Figure 3d an average SiO_2_ etching rate of 5.3 nm/min is extracted from these data.

## Conclusion

Though block copolymer micelle lithography (BCML) was previously proven to be a versatile tool to prepare ordered arrays of various nanoparticles, the idea to use these particles as a mask for subsequent etching processes such as RIE in order to fabricate nanopillars meets some serious problems in practice if large heights or aspect ratios of such pillars are needed. Obstacles to overcome are related to keeping the lateral position of the masking NP fixed and, even more serious, the nanomasks get significantly eroded by longer etching processes. The present work successfully demonstrated two ways of how to get around these obstacles:

- repeated OTMS stabilization and photochemical growth of nanomasks combined with a single etching step
- a single stabilization step followed by cyclic etching and re-growth of the photochemical mask

In silica the first method appears to be more suitable for preparing sub-30 nm sized pillars with an aspect ratio up to approximately 3:1. The second method is appropriate for pillar diameters (at half height) between 50 nm and 70 nm and extending their height to 700 nm, which delivers aspect ratios on the order of 10:1. The general principles described in the present work were experimentally realized based on hexagonally ordered arrays of Au NP that were prepared by BCML on top of the silica substrates.

Finally, it is worth noting that the silica nanopillars fabricated along the given recipes exhibit smooth vertical side walls without over-etching since the mask erosion problem is overcome by cyclic re-growing of the Au NP masks (Supporting Information File 1, Figure S3). Additionally, the residuals of the Au NP masks still present after the last etching step can be removed by using a commonly used gold etchant (Lugol’s solution) [20] without damaging the obtained SiO_2_ nanopillars.

## Experimental

**Materials used in the experiment:** Polystyrene-*block*-poly(2-vinylpyridine) diblock copolymer (PS(1850)-*b*-P2VP(900) was purchased from Polymer Source Inc., Canada. VLSI grade toluene was purchased from J. T. Baker, Netherlands. Gold(III) chloride hydrate (HAuCl_4_·H_2_O) and octadecyltrimethoxysilane (OTMS) were provided from Aldrich. Phthalatester mixture (product number: 1160) was from Cargille Labs, USA. All chemicals were of analytical grade. The 10 × 5 mm^2^ substrates were cut from n-Si with a 1 µm thick, thermally grown SiO_2_-layer (supplied by CrysTec, Berlin).

**Fabrication of hexagonally ordered Au NP-arrays:** A solution of PS-*b*-P2VP (20 g/L) in toluene was constantly stirred for a week. Spherical reverse micelles that were formed in the solution were loaded with metal precursor (HAuCl_4_·H_2_O) to a loading factor of 0.5 and stirred for another week. Substrates were dip-coated into the micellar solution transferring the loaded inverse micelles onto the SiO_2_-substrate surface. In order to reduce the precursor into metallic Au and to completely remove the polymeric species, substrates were treated in H_2_-plasma (90 min at 160 W, Technics Plasma 100-E, Munich).

**Chemical vapor deposition of OTMS onto Au decorated SiO_2_-substrates:** Au NP decorated SiO_2_-substrates were exposed to O_2_-plasma for 10 min to chemically activate the surface. The substrates were directly inserted into a desiccator, in which liquid OTMS was dropped in a glass platform inside. Due to the hygroscopic nature of the OTMS, the experiment was performed in a glove box at very low humidity (<5% RH). The sealed desiccator (0.2 mbar) was placed in an oven at 110 °C for 4 h for the self-assembly of OTMS. Finally, the substrates were rinsed with acetone and dried with nitrogen.

**Photochemical deposition of gold:** For the selective photochemical deposition of Au, a solution of phthalatester mixture and HAuCl_4_ at a concentration of 5 mM was prepared under ambient conditions. Phthalatester was chosen as solvent since it does not evaporate under UV-irradiation and has low absorption in the UV range. On each substrate 20 µL gold salt solution was homogeneously dispensed. The controlled seed growth of Au NPs was established under UV-irradiation (Osram Hg lamp; exposure wavelengths: 365 and 390 nm) provided by a mask aligner (Karl SUSS MJB 3 Mask UV 400). Afterwards, the substrates were subsequently cleaned in acetone and isopropanol and dried with nitrogen.

**Annealing:** Photochemically enlarged Au NP on SiO_2_-substrates were placed in ceramic boats and inserted into a tube furnace for annealing (Linn High Therm FRH-40/220/1250, Eschenfelden, Germany). The process was carried out at 720 °C for 4 h. Depending on the particle size, this time was further optimized.

**Anisotropic reactive ion etching (RIE):** A mixture of CHF_3_ and CF_4_ (20:2 sccm, 10 mTorr) plasma was applied to form uniform columnar structures on the substrates. Radio frequency (RF) power of 40 W, and a substrate temperature of 25 °C was used and the etching was carried out in Oxford PlasmaLab 80 Plus RIE device. The same process can also be directly employed for the etching of silicon and silicon nitride nanopillars with more cylindrical shaped pillars and improved aspect ratios.

**Characterization methods:** Both Au decorated and pillar patterned surfaces were investigated by high resolution scanning electron microscopy (Hitachi S5200 HRSEM, 30 kV) without additional conductive coating. The stage was either tilted by an angle of 30° or a special holder was used to acquire cross-section images. Particle size and density analyses were established by ImageJ 1.40g software. Self-assembly of OTMS was analyzed by a self-made contact angle measurement system with a droplet volume of typically 10 µL.

## Supporting Information

Supporting Information features complementary images and calculations of the stabilizing effect of an OTMS layer on the position of Au NP decorated substrates and cyclic fabricated high aspect ratio nanopillar arrays.

File 1 Complementary images and calculations.
