# Supplementary material for: Cyclic photochemical re-growth of gold nanoparticles: Overcoming the mask-erosion limit during reactive ion etching on the nanoscale
Source: Beilstein J Nanotechnol. 2013 Dec 12;4:886–94. doi: 10.3762/bjnano.4.100 (PMC3869346; doi:10.3762/bjnano.4.100)
Supplement: File 1 — Complementary images and calculations. [file Beilstein_J_Nanotechnol-04-886-s001.pdf]

Supporting Information

for

Cyclic photochemical re-growth of gold nanoparticles: Overcoming the mask-erosion limit during reactive ion etching on the nanoscale

Burcin Özdemir*, Axel Seidenstücker, Alfred Plettl and Paul Ziemann

Address: Institute of Solid State Physics, Ulm University, D-89069 Ulm, Germany

Email: Burcin Özdemir* - burcin.oezdemir@uni-ulm.de

* Corresponding author

Complementary images and calculations

Section S1: To demonstrate the stabilizing effect of an OTMS layer on the position of Au NP deposited by BCML on top of silica the following Figure S1 is added. There, in panel a) a HRSEM image is presented as obtained after photochemically size enhancing Au NP from the as-prepared 12 nm up to 30 nm *without* a stabilizing OTMS layer. Comparison to panel b) giving the result after identical size enhancement but performed *with* an OTMS layer immediately unveils that OTMS protects NP from coalescence and displacement.

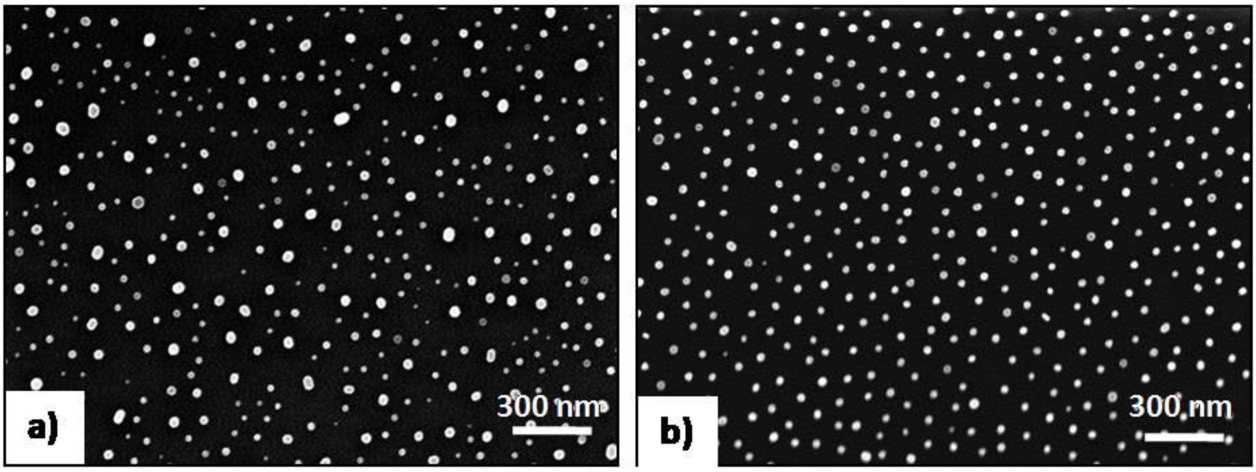

Figure S1: HRSEM images of photochemical growth of Au-NPs on SiO_2-substrate, a) without, and b) with stabilizing OTMS layer.

Section S2: As demonstrated in Section S1, if the substrate surface was not functionalized by OTMS, photochemical seeding process with subsequent annealing resulted in displacement and coalescence of the enlarged Au-NPs. In order to narrow down the possible reasons for the particle dislocations, experimental parameters such as solvent, UV-irradiation and metal precursor were analyzed separately. For the following experiments, the surface of the substrate was marked by a diamond tip to observe the changes in a defined location. Investigation of the particle movement was carried out by comparison of two HRSEM images taken from exact location before and after each experiment. For accurate analysis, the HRSEM-image shift and rotation of the images were taken into account. The coordinates of the particles in both images were determined by center of mass calculation using ImageJ software. A reference particle was chosen to calculate the shift.

Calculation of the particle dislocation:

Vector $\vec{K}$ was defined and calculated by using the coordinates of the reference particle in both images by Equation 1.

$$\vec{K} = \begin{pmatrix} d_x \\ d_y \end{pmatrix}, where\ d_x = x_2 - x_1\ and\ d_y = y_2 - y_1 \qquad (1)$$

3 data points were chosen for calculation of the rotation angle between images (Equation 2). The angle was calculated from several points and average for better accuracy.

$$\theta = \arccos(\frac{\overrightarrow{K_1}.\overrightarrow{K_2}}{|\overrightarrow{K_1}||\overrightarrow{K_2}|}) \qquad (2)$$

where $\overrightarrow{K_1}$the vector between two data is points on the image taken before and $\overrightarrow{K_2}$ is the vector after.

In addition, possible stretch on the SEM images was considered from the magnification. Combining the rotation angle θ and stretching factor in x (a_1) and y-directions (a_2), a rotation matrix R was formed (Equation 3).

$$R(\theta) = \begin{bmatrix} a_1 \cos\theta & -a_2 \sin\theta \\ a_1 \sin\theta & a_2 \cos\theta \end{bmatrix} \qquad (3)$$

The data points from the image taken after the experiment was transformed and rotated in order to match the original data points (Equation 4).

$$(D - \overrightarrow{a_0})^T . R(\theta) + \overrightarrow{a_0} + \vec{K} \qquad (4)$$

Applying that procedure to test whether wetting with phthalatester solvent and subsequent cleaning with acetone and isopropanol leads to NP displacements delivers the results presented in Figure S2. From the analysis shown in panel c) one excludes such displacements.

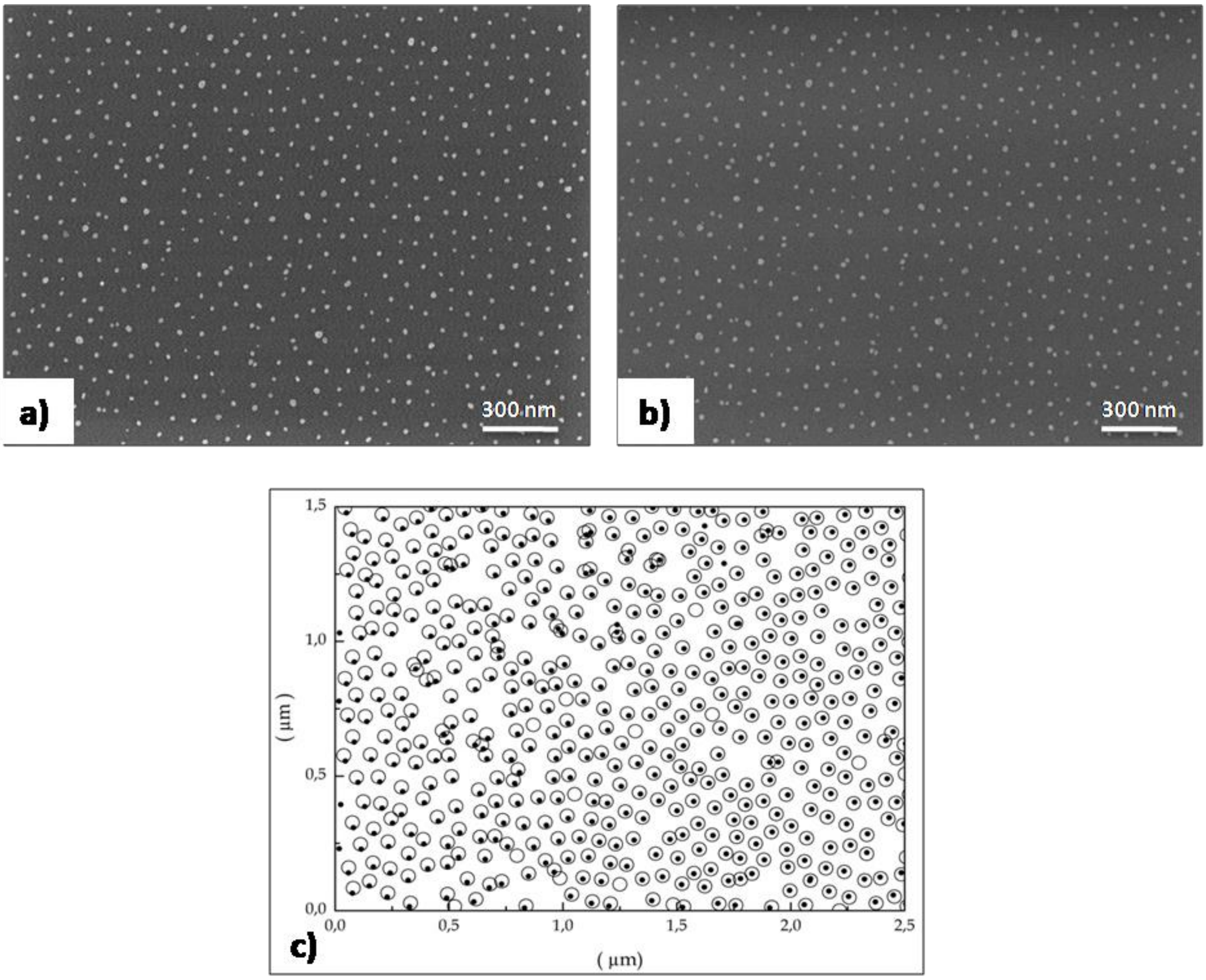

Figure S2: a) HRSEM images of photochemically grown particles (Ø ≈ 30 nm), wetted with phthalatester solvent and subsequently cleaned with acetone and isopropanol. a) Before and b) after the phthalatester solvent application. c) Comparison of the data points, particle locations are signed as before (circle) and after (dot) wetting.

Section S3: Additional HRSEM images documenting all growth cycles defined in Figure 5.

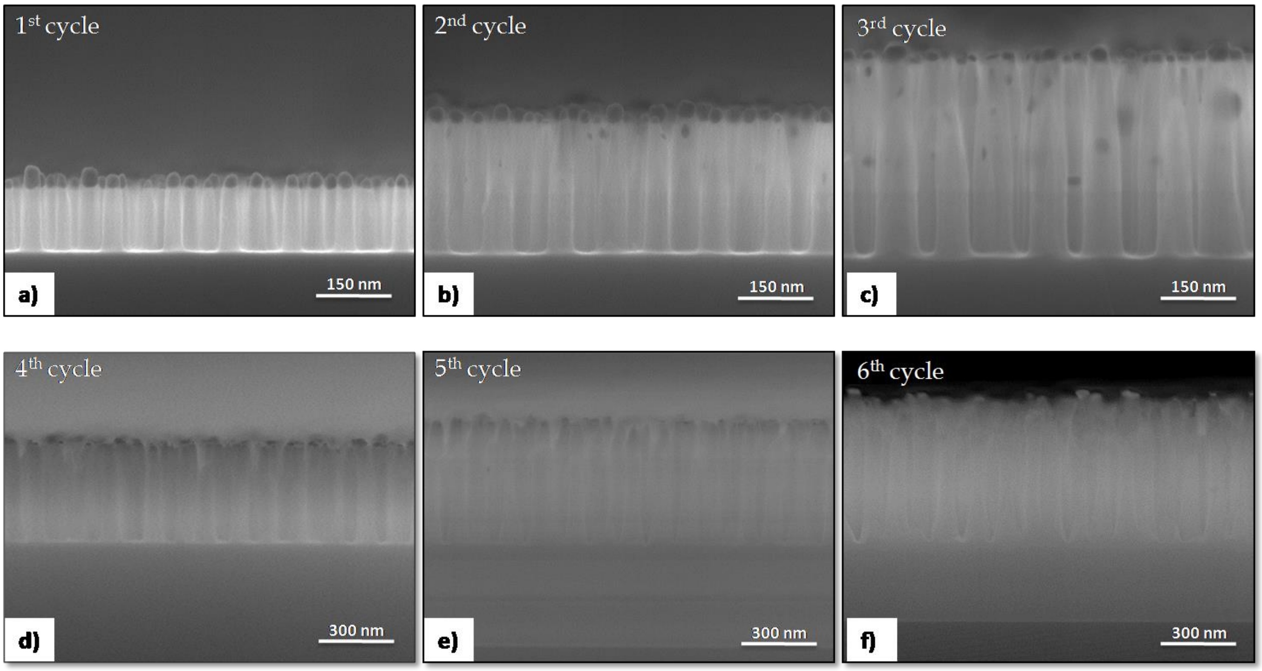

Figure S3: Cross-section HRSEM images of nanopillar arrays fabricated according to the schematics given in Figure 4 a)-f) after 1–6 cycles. After the 6th cyclic step, pillar height is enhanced to 680 nm after a total RIE reaction time of 120 min. Aspect ratio for well-defined columnar pillars is on average 10:1. For details see Table S1.

Table S1: Pattern geometries from Figure 5 and Figure S3 fabricated on the SiO_2.

	initial/final Au diameter (nm)	pillar height (nm)	FWHM of pillar diameter (nm)
initial	12.0 ± 2.1 / 9.2 ± 1.9	25.9 ± 1.9	14.6 ± 1.9
1st cycle	24.3 ± 4.2 / 14.9 ± 3.7	109.4 ± 4.9	23.4 ± 2.4
2nd cycle	24.2 ± 4.6 / 16.3 ± 2.6	204.9 ± 3.8	27.5 ± 2.7
3rd cycle	23.4 ± 3.9 / 18.5 ± 3.6	320.7 ± 4.9	37.0 ± 3.6
4th cycle	24.4 ± 3.8 / 16.2 ± 3.7	461.1 ± 6.3	56.9 ± 5,6
5th cycle	23.5 ± 4.4 / 14.5 ± 4.8	569.2 ± 4.9	61.9 ± 5,1
6th cycle	22.8 ± 4.1 / 12.2 ± 5.3	680.3 ± 8.7	66.6 ± 4.7
